# Simultaneous triple cancer of the esophagus, pancreas and rectum treated with multimodal strategies: a case report

**DOI:** 10.1186/s40792-020-01035-0

**Published:** 2020-10-02

**Authors:** Hiroki Imamura, Hajime Hirose, Shunji Endo, Yasuji Hashimoto, Masashi Takeda, Shinya Kidogami, Yukako Mokutani, Tomoya Kishimoto, Shinichi Yoshioka, Shigeyuki Tamura, Yo Sasaki

**Affiliations:** 1 Department of Surgery, Yao Municipal Hospital, 1-3-1, Ryuge-cho, Yao-shi, Osaka, Japan; 2 Department of Pathology, Yao Municipal Hospital, 1-3-1, Ryuge-cho, Yao-shi, Osaka, Japan

**Keywords:** Simultaneous triple cancer, Esophagus, Pancreas, Rectum

## Abstract

**Background:**

Due to the development of diagnostic imaging technology, we have increased chance of detecting multiple primary cancers. However, simultaneous triple cancer is still a very rare finding whose frequency is not yet known. Treatment of simultaneous triple cancer is a clinical challenge because it requires multimodal strategies including surgery, chemotherapy and radiotherapy.

**Case presentation:**

Here, we present the case of a 74-year-old male with triple cancer involving esophageal and pancreatic cancer, and rectal carcinoma. Each cancer was surgically resectable, but simultaneous resection of all cancers seemed to cause too much surgical stress for the patient. First, we performed a laparoscopic Hartmann’s operation for rectal cancer to minimize the risk of postoperative complications. Then treatment for pancreatic cancer was initiated by administering neoadjuvant chemotherapy with gemcitabine plus nab-paclitaxel. The pancreatic tumor shrank in size, so pancreatoduodenectomy was performed. We chose S-1 as adjuvant chemotherapy. The esophageal cancer showed regression during the treatment of the other two cancers, likely because the chemotherapeutic agents administered for pancreatic cancer had some effect on the esophageal cancer. Definitive chemoradiotherapy was selected instead of esophagectomy because the patient had already undergone two major surgeries. The patient is still alive nine months after the whole course of treatment with no sign of recurrence.

**Conclusions:**

The treatment of triple cancer requires an elaborate strategy to determine which cancer has to be dealt with first and which can be treated later. An aggressive multimodal treatment strategy may be an important option for a patient with triple cancer.

## Background

Due to the development of diagnostic imaging technology, we have increased chance of dealing with multiple primary cancers. However, simultaneous triple cancer is still a very rare finding whose frequency is not yet known. Treatment of simultaneous triple cancer is a clinical challenge because it requires multimodal strategies including surgery, chemotherapy and radiotherapy. Treatment can be more challenging when triple cancer involves malignancies requiring invasive surgical procedures such as pancreatic or esophageal cancer.

## Case presentation

**Fig. 1:**
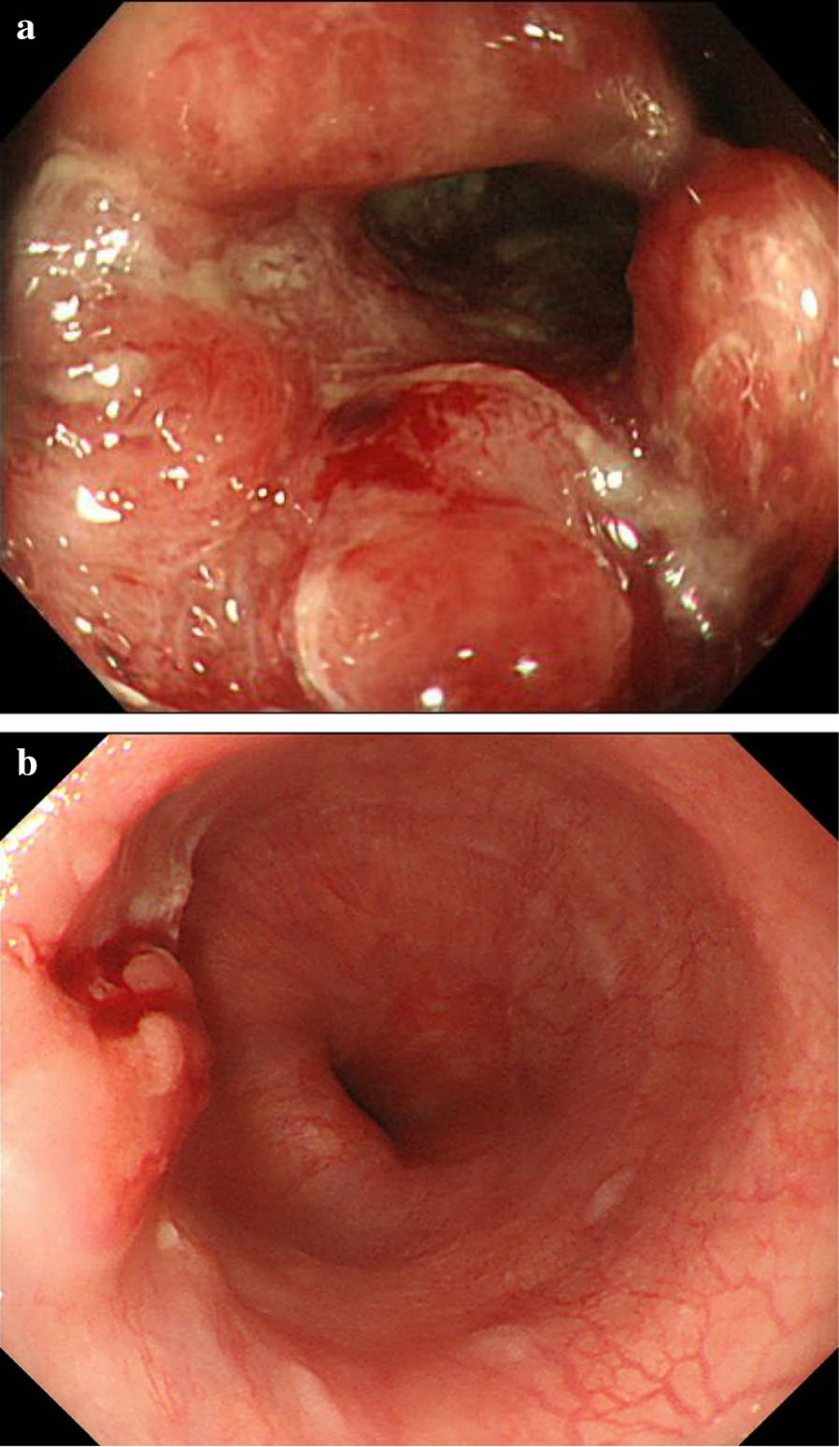
Endoscopic findings at presentation. **a** Colonoscopy revealed a circumferential rectal tumor 7 cm from the anal verge. **b** Esophagogastroduodenoscopy showed type 0-I + IIb esophageal cancer that was 36–39 cm from the front teeth

**Fig. 2:**
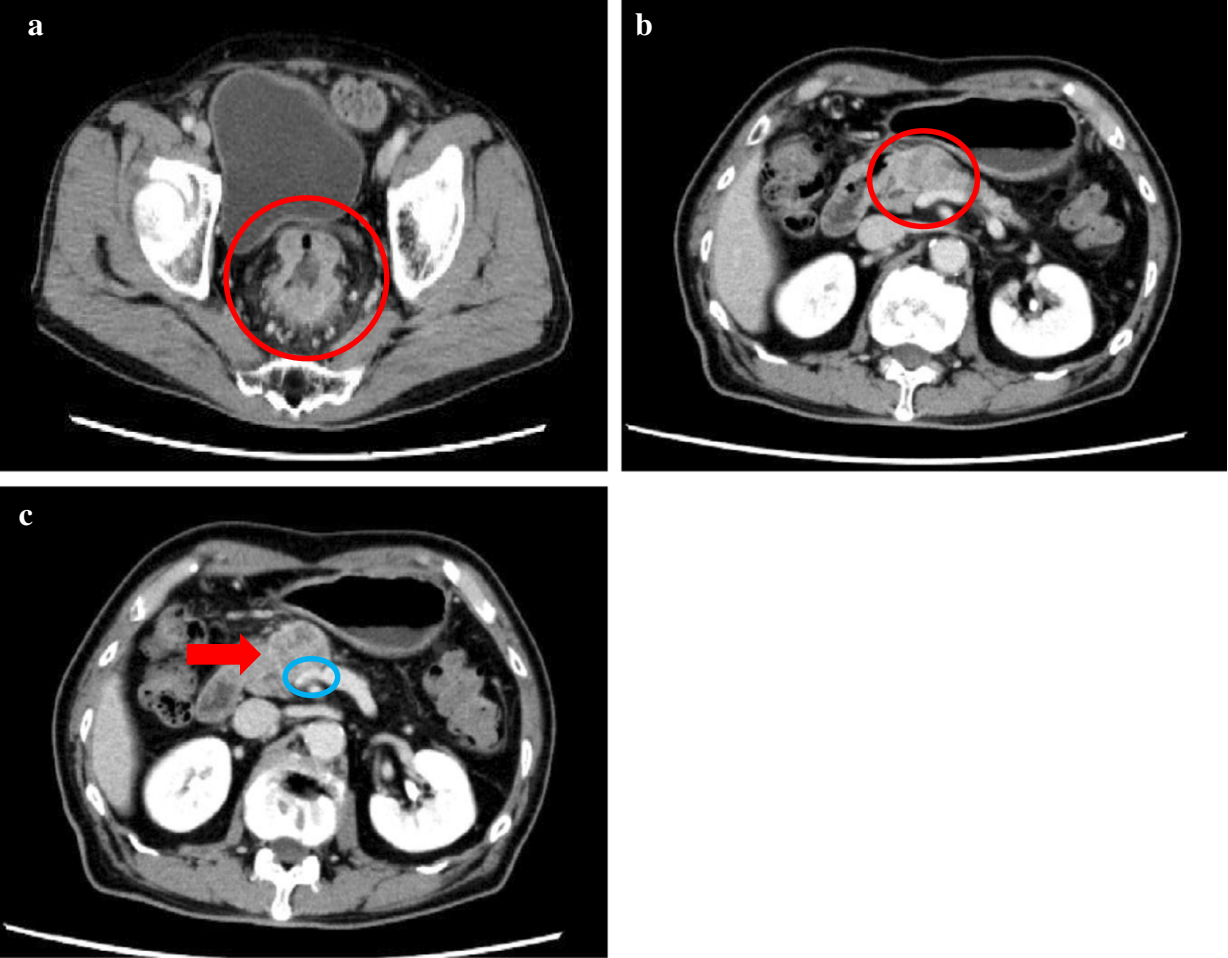
Computed tomography findings at presentation. **a** Preoperative contrast-enhanced computed tomography showed a clearly enhanced rectal lesion in Ra (red circle) with no lymphadenopathy or distant metastasis. **b** Contrast-enhanced computed tomography also revealed a poorly enhanced tumor 3 cm in size in the pancreatic head (red circle). **c** The pancreatic tumor (red arrow) widely abutted the superior mesenteric vein (blue circle)

A 74-year-old man was referred to our hospital for the examination of diarrhea and hematochezia lasting for 3 months. A colonoscopy revealed a circumferential advanced type 2 rectal cancer at 7 cm from the anal verge (Fig. 1a). The colonoscope managed to pass through the tumor, but the tumor made the rectal lumen so narrow that it could safely be assumed that the rectum would be obstructed within a few months. The pathological result of the biopsy was moderately differentiated adenocarcinoma. A contrast-enhanced computed tomography scan (CE-CT) showed clearly enhanced rectal lesion in the upper rectum (Ra) with no lymphadenopathy or distant metastasis (cT3N0M0, cStage IIa, Japanese Society for Cancer of the Colon and Rectum (JSCCR), 3rd edition [1]; cT3N0M0, cStage IIA, Union for International Cancer Control (UICC), 8th edition [2]) (Fig. 2a).

CE-CT also revealed a poorly enhanced tumor 26 mm in size in the pancreatic head (Fig. 2b). Pancreatic cancer that invaded the anterior membrane was suspected. The tumor widely abutted the superior mesenteric vein (Fig. 2c), implying that the tumor was borderline resectable [3]. No lymphadenopathy or distant metastasis was detected by CE-CT (cT3N0M0, cStage IIA, Japan Pancreas Society (JPS), 4th edition [4]; cT3N0M0, cStage IIA, UICC 8th [2]).

An esophagogastroduodenoscopy (EGD) was performed to assess malignancy in the upper gastric tract, revealing the presence of type II esophageal carcinoma 36–39 cm from the front teeth possibly with muscularis propria invasion (Fig. 1b). No lymphadenopathy or distant metastasis was detected by CE-CT (cT2N0M0, cStage II, Japan Esophageal Society (JES), 11th edition [5]; cT2N0M0, cStage II, UICC 8th [2]). The pathological diagnosis of the biopsied specimen was squamous cell carcinoma.

**Fig. 3:**
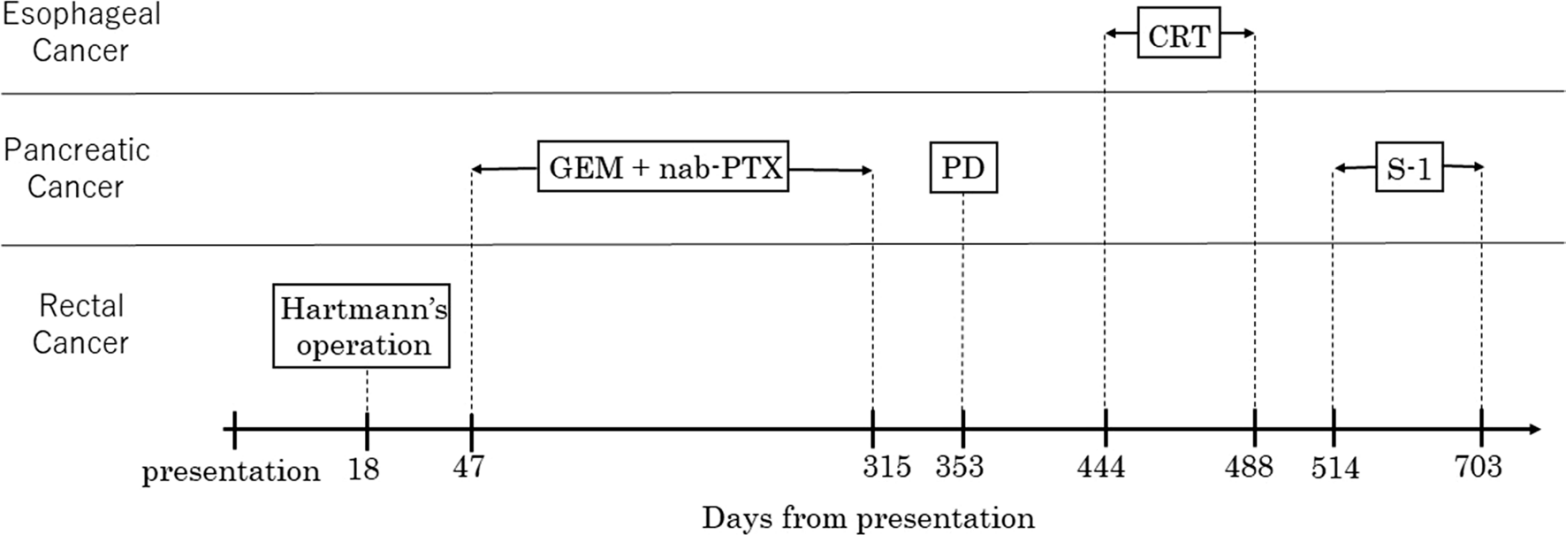
The outline of whole course of treatment. *GEM* gemcitabine, *nab-PTX* nab-paclitaxel, *PD* pancreatoduodenectomy, *CRT* chemoradiotherapy

**Fig. 4:**
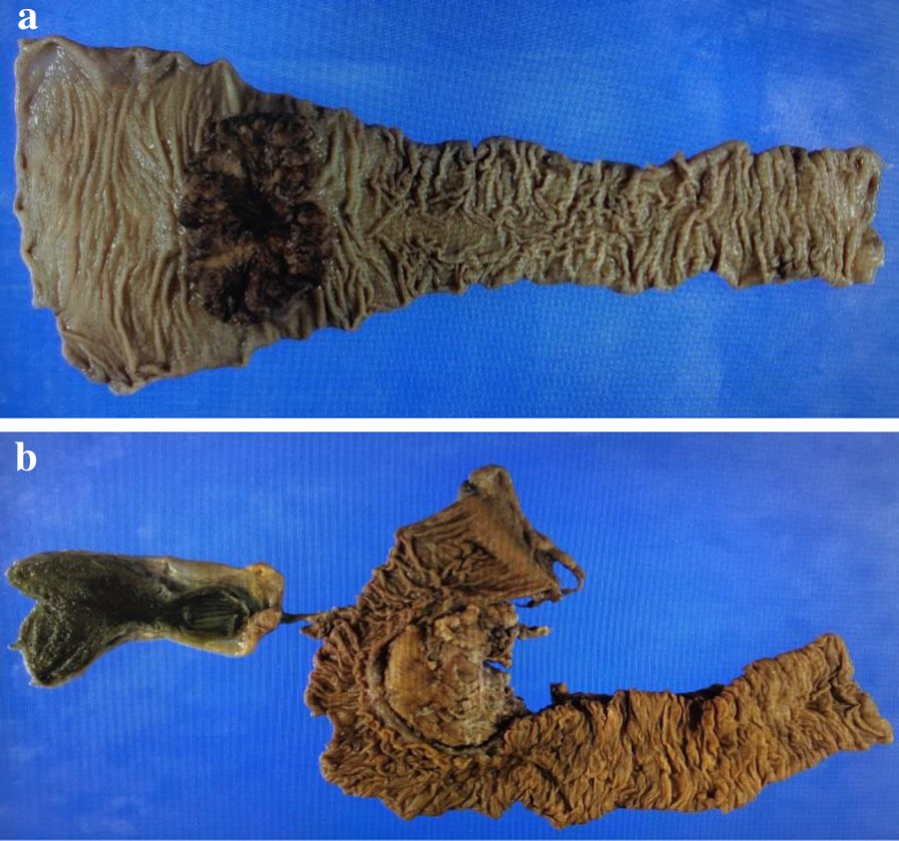
Surgical specimens. **a** The rectal tumor was resected by Hartmann’s operation. **b** The pancreas tumor was resected by pancreatoduodenectomy

The clinical diagnosis was simultaneous advanced triple cancer involving esophageal and pancreatic cancer, and rectal carcinoma. Each tumor was surgically resectable, but the simultaneous resection of the three tumors was likely to cause too much stress. Although pancreatic cancer was the prognostic determinant for the patient, rectal obstruction had to be prevented to initiate treatment for pancreatic cancer. Therefore, we chose the rectal cancer as our first target of treatment. The outline of the whole course of treatment is described in Fig. 3. We performed a laparoscopic Hartmann’s operation to minimize the likelihood of postoperative complications. The operation time was 424 min, and the blood loss was minimal. The pathological result was moderately differentiated adenocarcinoma, Ra, pT3N0M0, pStage IIa (JSCCR 3rd [1]); pT3N0M0, pStage IIA (UICC 8th [2]), R0 resection (Fig. 4a). The postoperative clinical course was remarkable for a portal vein thrombus (PVT) detected by CE-CT, which was performed on the 5th day after surgery to evaluate abnormally elevated liver enzymes. Oral edoxaban at 30 mg/day was initiated, after which the levels of liver enzymes gradually improved. The patient was discharged on the 19th day after surgery.

**Fig. 5:**
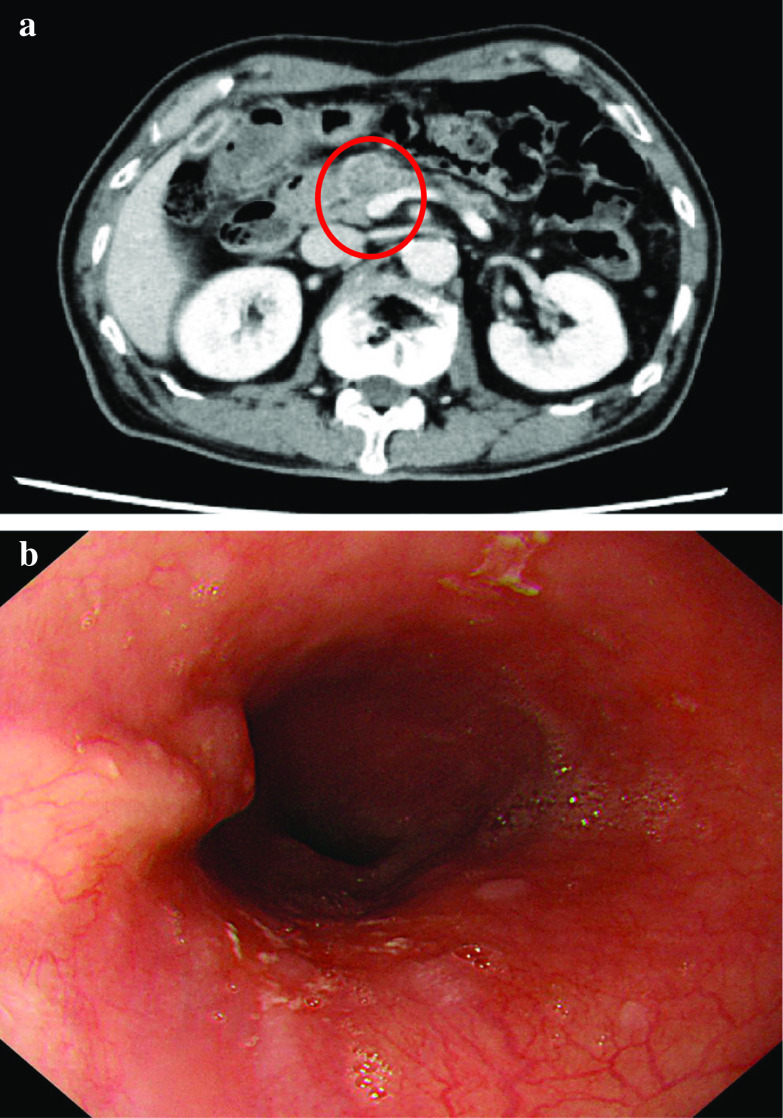
Therapeutic assessments after neoadjuvant chemotherapy for treatment of pancreatic cancer. **a** Computed tomography showed regression of the pancreatic tumor (red circle) in size from 26 to 18 mm after neoadjuvant chemotherapy. **b** The regression of esophageal cancer was endoscopically verified for its invasion into the muscularis mucosae after neoadjuvant chemotherapy for treatment of pancreatic cancer

The pancreatic tumor was our next target because the prognostic determinant for this patient was assumed to be pancreatic cancer rather than esophageal cancer. The borderline resectability of the tumor and the possibility of PVT being a tumor embolism led us to the initiation of neoadjuvant chemotherapy (NAC) for pancreatic cancer. The regimen of gemcitabine plus nab-paclitaxel was started with the expectation of its being effective for both esophageal and pancreatic cancer, and it was administered for 10 courses. The duration of NAC was prolonged due to the patient’s preference. After NAC, PVT disappeared, and the main tumor shrank from 26 to 18 mm in diameter (Fig. 5a). No lymphadenopathy or distant metastasis appeared on a follow-up CE-CT. An EGD was also performed to assess to what extent NAC, which was performed for treatment of pancreatic cancer, was effective for the treatment of esophageal cancer. Esophageal cancer showed regression in terms of its invasion into the muscularis mucosae (ycT1aN0M0, ycStage 0, JES 11th [5]; ycT1aN0M0, ycStage I, UICC 8th [2]) (Fig. 5b). The clinical effect of NAC on the pancreatic and esophageal cancers resulted in partial response and stable disease based on the RECIST classification version 1.1 [6], respectively. As a result of the preoperative diagnosis of pancreatic cancer (ycT2N0M0, ycStage IB (JPS 4th [4]) and ycT1cN0M0, ycStage IA (UICC 8th [2])), the pancreatoduodenectomy (PD) with portal vein resection and reconstruction was performed. The operation time was 498 min, and the blood loss was 780 ml. The pathological result indicated invasive ductal carcinoma of the pancreas (ypT1cN0M0, pStage IA (JPS 4th [4]) and ypT1cN0M0, ycStage IA (UICC 8th [2])), R0 resection (Fig. 4b). The patient did not develop any complications until discharge on the 24th day after surgery. The patient was administered S-1 as adjuvant chemotherapy for 6 months, which is also commonly used as a part of the chemotherapy regimen for esophageal cancer. S-1 was initiated 26 days after the completion of definitive chemoradiotherapy (CRT) for esophageal cancer.

**Fig. 6:**
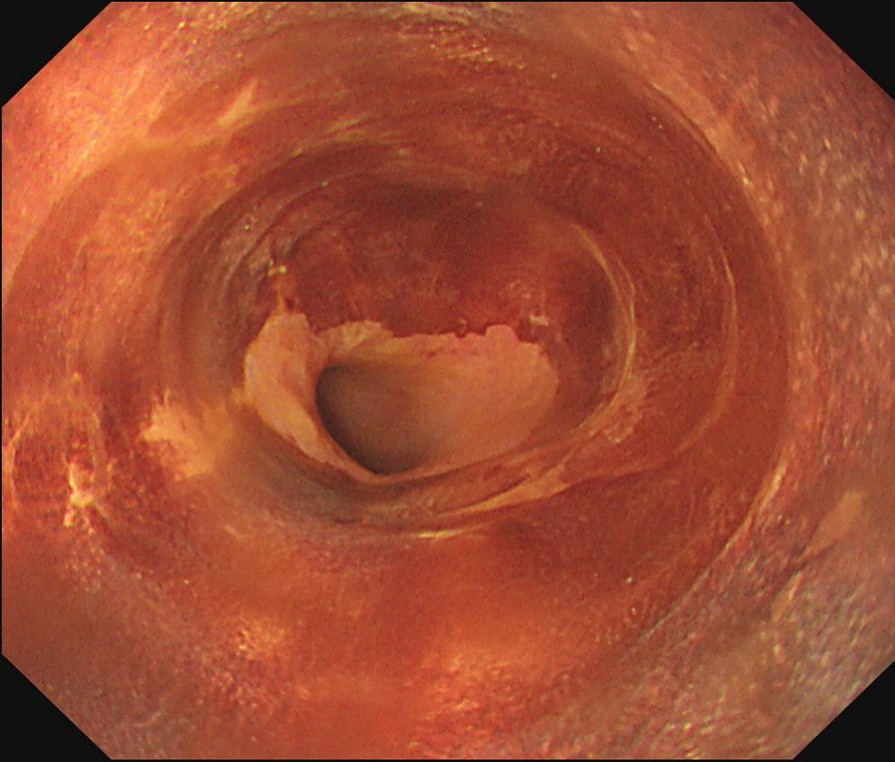
Endoscopic findings after chemoradiotherapy for esophageal cancer treatment. Complete disappearance of the tumor was verified endoscopically. The unstained area remained after iodine staining, but malignant cells were not detected on biopsy

For the esophageal lesion, we decided to perform CRT, as surgical resection might have been too invasive after the two previous surgeries. Chemotherapy was started with cisplatin plus fluorouracil, and the amount of radiation was 59.4 Gy/33Fr. CRT was continued for 44 days. A follow-up EGD showed the complete disappearance of the tumor. The treatment effect caused a complete response according to the RECIST classification version 1.1 (Fig. 6). The patient is alive with no recurrence nine months after the entire course of treatment.

## Discussion

MPC is a rare finding whose frequency is reported to vary from 2.4 [7] to 18.4% [8]. The variation is caused partly by the development of diagnostic imaging and partly by the presence of multiple definitions of MPC. According to Warren and Gates, MPC is defined by the following three criteria: each of the tumors must be histologically different; each should involve different organs; and the possibility of one cancer being a metastasis of the other must be excluded [9]. MPC is generally divided into two categories according to the timing of tumor detection: simultaneous, which occurs when tumors are diagnosed simultaneously or within a 6-month interval; metachronous, which occurs when the diagnosis interval is longer than 6 months [10]. Our case involved the almost simultaneous diagnosis of three tumors that were histologically distinct from each other and involved different organs, the esophagus, pancreas, and rectum with no metastasis. This means that our case involves simultaneous triple cancer (STC) of the esophagus, pancreas and rectum.

There have been some case reports on STC to date, but none of them have referred to the frequency of STC, indicating that STC is an extremely rare condition. Moreover, as far as we could determine, our case is the first report on STC involving esophageal and pancreatic cancer, and rectal carcinoma.

Treatment for STC is a clinical challenge because treatment often requires multimodal therapy, including not only surgery but also chemotherapy and radiotherapy. In addition, simultaneous treatment of all the existing tumors sometimes places too much stress on patients, so we must carefully consider which tumor should be dealt with first and which tumor can be treated later. In developing a treatment strategy, a useful theory is the one advocated by Boute [11]: the prognosis of MPC is not determined by its simultaneous nature, but by the independent progression of each disease. When we separately assessed the prognosis of each tumor in our case, cStage II esophageal cancer, cStage IIA pancreatic cancer, and cStage IIa rectal cancer were found to have a 5-year overall survival rate of 62.7% [12], approximately 10% [13], 84.6% [14], respectively. From a prognostic point of view, pancreatic cancer was the first target, and the initiation of NAC for pancreatic cancer was warranted because of its borderline resectability. However, safe and effective NAC would have been impossible if the rectum had become obstructed by rectal cancer. Therefore, we decided to treat the rectal cancer first. The construction of colostomy was not our option because cStage IIA rectal cancer is indicative of surgical resection and we assumed the patient was tolerable of surgery. Considering that the anastomosis level was supposed to be less than 5 cm from the anal verge and also that the operation took relatively a long time, we chose a laparoscopic Hartmann’s operation rather than low anterior resection to avoid the risk of anastomosis leakage. It is reported that both low anastomosis level and operation time were a significant predictive value of anastomotic leakage [15]. It is widely accepted that construction of diverting ileostomy can reduce the rate of anastomotic leakage. Hamabe et al. report that in high risk male patients in whom tumor is located within 7 cm from anal verge or preoperative chemotherapy was performed, the rate of anastomotic leakage can be significantly reduced to be 10.7% by creating diverting ileostomy [16]. However, if the anastomotic leakage had occurred in our patient, who had esophageal and pancreatic cancer left untreated, his prognosis would have been much worse because the treatment for the remaining two cancers would have been impossible to start. That was why we chose Hartmann’s operation which requires no anastomosis. The postoperative course was worth paying attention because PVT appeared on postoperative day 5. We considered two causes that could result in PVT: the long operation time for Hartmann’s procedure, and the pancreatic cancer. PVT after colorectal surgery is rare. Allaix et al. report that 3.5% of the patients undergoing colorectal surgery experience postoperative PVT and that operation time longer than 220 min is one of the risk factors for PVT [17]. PVT caused by pancreatic cancer seems rarer. Yamato et al. report that there had been only 18 case reports of PVT associated with pancreatic cancer from 1985 to 2007 [18]. In our case, considering that hepatic enzyme level improved after administration of edoxaban, the cause of PVT was more likely the long operation time. After the rectal cancer was successfully resected, our next step was to initiate NAC for pancreatic cancer. The ultimate objective of NAC was to maximize the potential for R0 resection because, as Pavan Tummala et al. reported, R0 resection for pancreatic cancer yields a better prognosis [19]. Regarding adjuvant chemotherapy for resected pancreatic cancer, S-1 is reported to be effective in improving the prognosis [20]. After the whole course of treatment for rectal and pancreatic cancer was completed, the esophageal cancer showed regression. This may be because the chemotherapy regimen administered for pancreatic cancer was partially effective for esophageal cancer. Gemcitabine and nab-paclitaxel are sometimes reported to be effective for advanced esophageal cancer [21, 22]. The selection of a chemotherapeutic regimen is considered to be especially important for a patient with MPC because chemotherapy agents effective for one cancer may benefit the treatment of the other coexisting cancers. From that point of view, our choice of gemcitabine plus nab-paclitaxel and S-1 was beneficial for our patient because these agents were able to exert simultaneous therapeutic effects on multiple cancers. According to the Japanese guidelines for esophageal cancer, definitive CRT or surgery is indicated for cStage II esophageal carcinoma depending on the patient’s condition [23]. Although the Japanese guidelines are based on weak evidence that recommends surgery-based therapy over chemoradiotherapy for patients with cStage II or III esophageal cancer as long as patients are tolerable for surgery [23], we considered that esophagectomy carried a great risk for our patient because he had already undergone two major surgeries. In addition, reconstruction after esophagectomy would not be easy because the gastric and jejunal graft conduits were almost completely unavailable because of the two surgeries the patient had previously undergone. Reconstruction with the right hemicolon might have been technically possible, but it also carried a great surgical risk. This is why we chose to use definitive CRT for this patient. The patient is now alive nine months after the whole course of treatment with no sign of recurrence. If local recurrence occurs in the esophagus, salvage surgery may be one of the treatment options, depending on the patient’s condition.

## Conclusion

This case is remarkable in that it involved three synchronous cancers, and definitive treatment was successfully achieved for all three cancers. STC is a clinically demanding condition, but aggressive treatment may be effective if it is indicated.

## Data Availability

Yao Municipal Hospital.

## Abbreviations

MPC: Multiple primary cancer
CE-CT: Contrast-enhanced computed tomography
JSCCR: Japanese Society for Cancer of the Colon and Rectum
JPS: Japan Pancreas Society
EGD: Esophagogastroduodenoscopy
JES: Japan Esophageal Society
NAC: Neoadjuvant chemotherapy
PD: Pancreatoduodenectomy
CRT: Chemoradiotherapy
STC: Simultaneous triple cancer
